# Small molecule detection with aptamer based lateral flow assays: Applying aptamer-C-reactive protein cross-recognition for ampicillin detection

**DOI:** 10.1038/s41598-018-23963-6

**Published:** 2018-04-04

**Authors:** Lars Kaiser, Julia Weisser, Matthias Kohl, Hans-Peter Deigner

**Affiliations:** 1 Furtwangen University, Institute of Precision Medicine, Jakob-Kienzle-Straße 17, 78054 Villingen-Schwenningen, Germany; 2 Institute of Pharmaceutical Sciences, University of Freiburg, Albertstraße 25, 79104 Freiburg i. Br., Germany; 3 Fraunhofer Institute IZI, Leipzig, EXIM Department, Schillingallee 68, D-18057 Rostock, Germany

## Abstract

Aptamer-based lateral flow assays (LFAs) are an emerging field of aptamer applications due to numerous potential applications. When compared to antibodies, potential advantages like cost effectiveness or lower batch to batch variations are evident. The development of LFAs for small molecules, however, is still challenging due to several reasons, primarily linked to target size and accessible interaction sites. In small molecule analysis, however, aptamers in many cases are preferable since immunogenicity is not required and they may exhibit even higher target selectivity. We report the first cross-recognition of a small molecule (ampicillin) and a protein (C-reactive protein), predicted by *in-silico* analysis, then experimentally confirmed - using two different aptamers. These features can be exploited for developing an aptamer-based LFA for label-free ampicillin detection, functioning also for analysis in milk extract. Most importantly, the principal setup denotes a novel, transferable and versatile general approach for detection of small molecules using competitive LFAs, unlikely to be generally realized by aptamer-DNA-binding otherwise.

## Introduction

Lateral flow assays (LFAs) are simple, fast and cost-effective test systems with high suitability for point-of-care testing (POCT). LFAs are widely applied, including detection of pathogens, food allergens or protein biomarkers of several medical conditions^1–3^. They are used for qualitative and to some extent for quantitative monitoring in several areas, including food production or non-laboratory environments^3^. LFAs usually employ antigen-antibody binding reaction, combined with lateral fluid flow through a membrane. Antibodies, however, have several limitations; for example, antibodies cannot be raised against toxic or non-immunogenic targets^3^. Low-molecular-weight (LMW) compounds, like metabolites or drugs, lacking intrinsic immunogenicity, make it difficult to raise specific antibodies against them. Even though, some antibodies exist against LMW compounds and also have been implemented in LFA applications, their selectivity often remains low, thus limiting LFA applications, which can be overcome by aptamers as probes^4–6^.

Aptamers are single-stranded DNA or RNA molecules, which can form 3D motifs and selectively bind to corresponding targets. They are comparable to monoclonal antibodies; properties, however, are quite different, since they belong to a different class of biomolecules. The main advantage of aptamers is that, in principle, they can be generated against any desired target, toxic small molecules, non-immunogenic targets or even single molecules, which does not apply to antibodies. Another advantage of aptamers is, that they can be easily and reversibly denaturized by heat or chemicals, due to their robust phosphodiester backbone, a feature which antibodies do not possess. Aptamers are cheap in production, own an extreme low batch-to-batch variation and possess a longer shelf life, compared to antibodies.

In recent years, many reports, using aptamers as probes in LFAs have been published. The targets mainly comprise proteins like thrombin^7^ and whole cells like bacteria^8^ or eukaryotic cells^9^. Few reports on the detection of LMW compounds using aptamer based LFAs, however, have been published. To the best of our knowledge, the reports from Zhang^10^, Özalp^11^, Fischer^12^, Liu^13^, Mazumdar^14^, Wang^15^ and Shim^16^ are the only reported aptamer based LFA systems for detection of small molecules up to date, more precisely, for detection of adenosine triphosphate (ATP), *p*-aminohippuric acid, adenosine, cocaine, lead, Ochratoxin A, Aflatoxin B1, E2 and BPA. Problems of aptamer selection are, however, inversely correlated to the size of the target molecule and its associated interaction sites^17^.

For the adaption of LFAs for small molecule detection, several key issues need to be considered, mostly regarding the working principle of LFAs. They are typically set up as sandwich or competitive formats, comparable to an Enzyme-Linked ImmunoSorbent Assay (ELISA). In case of the sandwich format, two probes are needed for target immobilization and detection. This is feasible in the case of larger targets (e.g. proteins), exhibiting several epitopes for probe binding. Small molecules, however, frequently exhibit only one site for probe binding, rendering this approach inapplicable. Ways to overcome this problem include splitting the aptamer in two parts, for instance as shown for an ATP aptamer^18^. This approach, however, may not be transferred in general to other targets. Another LFA set-up is the competitive format, in which the native analyte competes with an on strip immobilized analyte or a ssDNA strand, complementary to the aptamer^3^. Both approaches possess several issues. In the case of immobilizing the analyte, labelling can change the conformation of the small molecule in a way that the aptamer cannot bind to the target anymore or block the required binding site^19^. Furthermore, hybridization-based systems on LFAs are generally not preferable since kinetics of hybridization and lateral flow mass transport can hardly be matched; hybridization has been reported to require a minimum of 500 seconds in a microarray experiment, whereas the sample solution can quickly pass the whole length of the membrane within 600 seconds in LFAs^3,20^. Thus, LFA utilizing DNA binding of aptamer via hybridization would usually require reaction prior to the flow. Facing such difficulties, the *de-novo* development of an aptamer based LFA for small molecule detection remains a challenge.

We have chosen ampicillin as a small molecule model for developing an aptamer based LFA. It is an antibiotic substance, belonging to the penam class of beta lactam antibiotics. It is widespread in the use against medically important microorganisms, such as *streptococcus*, *salmonella*, *enterococcus*, *neisseria*, *bacillus anthracis*, or *borrelia*^21^. Allergic reactions towards penicillins, including ampicillin, are quite common and thus need to be considered. Reactions can include exanthema, breathing difficulties or even anaphylactic shock, showing the relevance of ampicillin in clinical and non-clinical areas. Additionally, allergic reactions are not always directed against all beta-lactams, but selective allergic reactions against ampicillin have been reported as well, making the determination of the respective antibiotic, for instance in food, important^22^. Maximum residue limits for ampicillin (e.g. 4 µg/kg in milk, 50 µg/kg in animal tissues, EU Regulation no 37/2010) are established by several regulatory authorities and the current quantitative methods for antibiotics include microbiological assays and high-pressure liquid chromatography (HPLC) analyses. Both approaches, however, are time consuming and are either of low specificity (microbiological assays) or need expensive sample preparation (HPLC)^23,24^. Thus, there is a need for a simple, cheap and more rapid ampicillin detection method.

In the parent paper, we report on a cross-reaction of an aptamer^23^ for ampicillin with C-reactive protein and vice versa on a C-reactive protein aptamer^25^ binding also ampicillin. Even though cross-reactions of aptamers between different protein variants have been published (e.g.^26^), to the best of our knowledge, this is the first report on a cross-recognition between an LMW compound selective aptamer with a protein. Based on this cross-recognition, we developed a competitive LFA, as a proof of principle. This demonstrates a new and versatile approach for the quantification of LMW compounds by LFAs.

## Results

### Sequence similarities of the two aptamers

**Figure 1:**
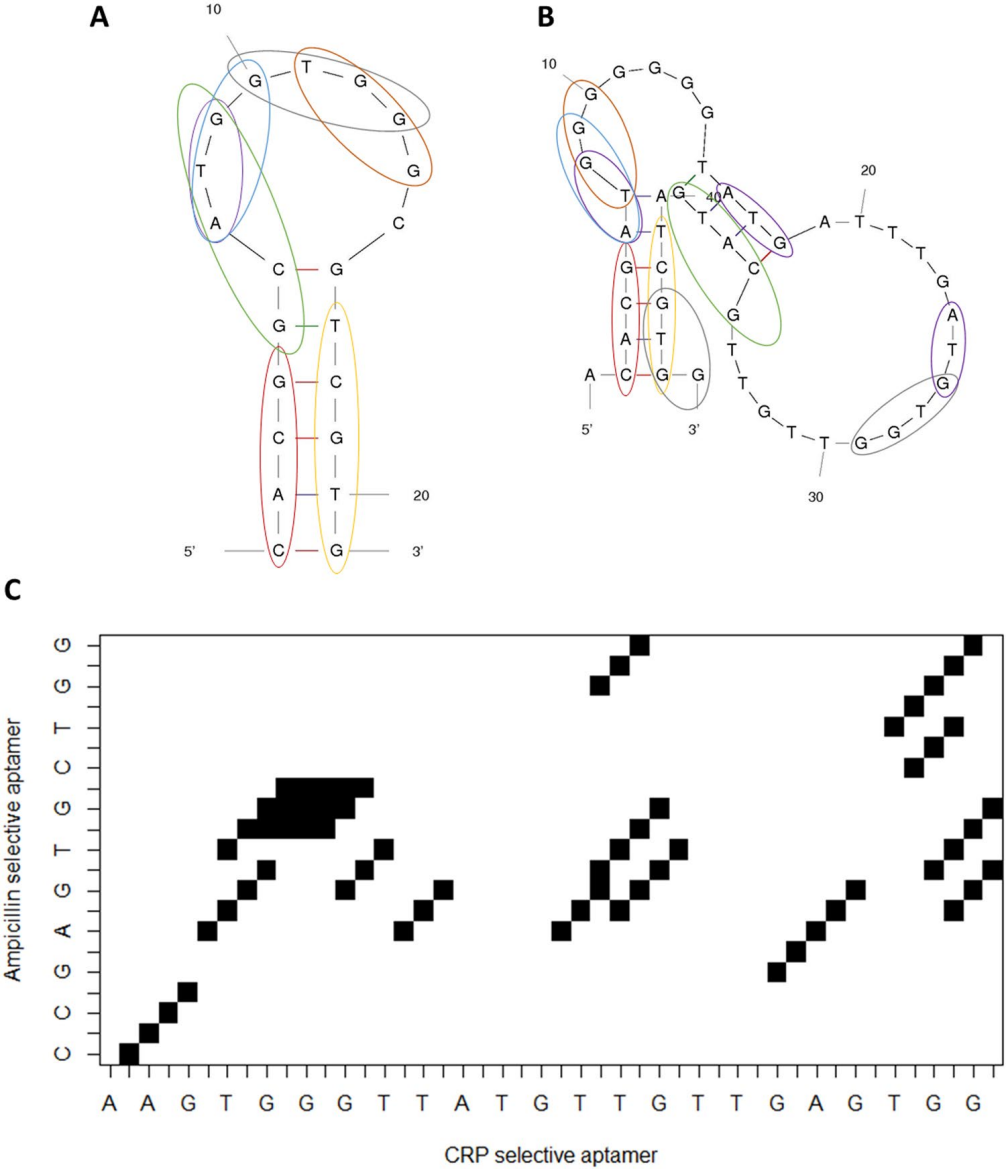
Comparison of the sequence identities of the used ampicillin aptamer (**A**) and the CRP aptamer (**B**). The different colors stand for the specific sequences, however, the ATG sequence (purple) occurs several times in the CRP aptamer sequence (not all identities are shown). Sequence identities with more than two nucleotides in a row of the ampicillin aptamer (y axis) and the CRP aptamer (x axis) are shown in the dot blot (**C**). Dot blot was created using a by M. Kohl modified function from the package “seqinr”.

By using an *in-silico* sequence comparison approach, we discovered the C-reactive protein aptamer^25^ sharing significant sequence similarities with the ampicillin aptamer^23^. Although both aptamers differ strongly at first glance, such as in size (23 nucleotides vs. 46 nucleotides) and for the folding structure as predicted by the Mfold software (see Fig. 1A and B), they share partial sequence overlap. Although, both targets belong to distinct biomolecule classes and a cross-reactivity appeared unlikely, both aptamers displayed partial sequence overlap as shown in Fig. 1. The most obvious sequence homology is found at the stem region of both aptamers (highlighted in red and yellow). Furthermore, some of the conserved regions at the loops (especially black and orange) may be crucial for target binding. Ban and coworkers reported that the TGG sequence is conserved in most ampicillin aptamers identified using the SELEX process^23^, however, FAM-labeled aptamers still remain bound to CRP-biotin after alteration of the TGG domain in both aptamers (Electronic Supplementary Material Fig. S3B). Both aptamers display several regions sharing sequence identity, indicating that both aptamers might bind to the same targets.

### Cross-recognition of both targets by both aptamers

**Figure 2:**
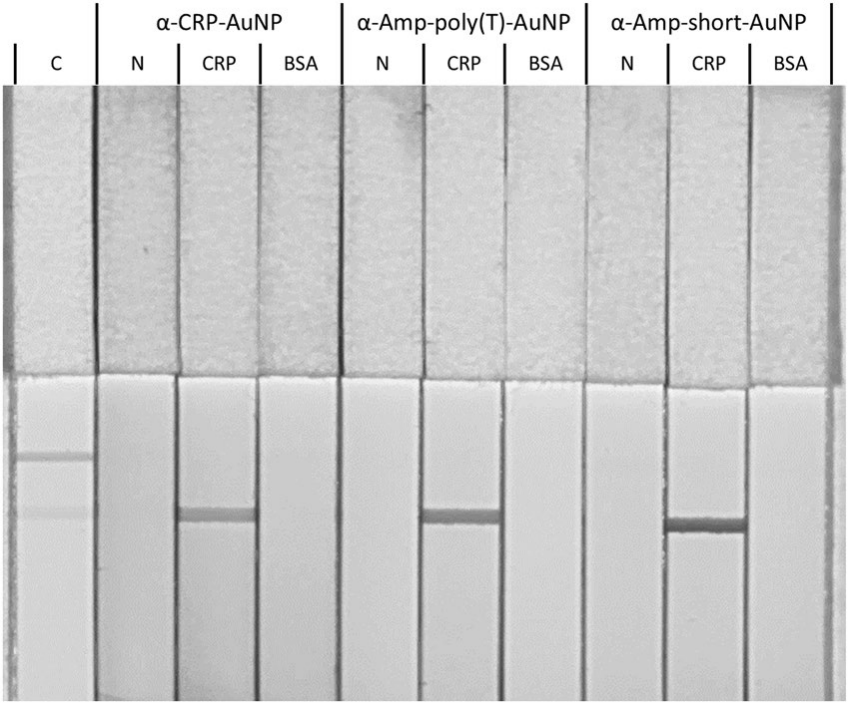
Lateral Flow Assay, using different aptamer-AuNP conjugates (α-CRP-AuNP, α-Amp-poly(T)-AuNP and α-Amp-short-AuNP) in combination with different biotinylated proteins (CRP and BSA), as well as absence of biotinylated proteins (N). 40.5 µg biotinylated protein was used per strip, a control test stripe (**C**) indicates the test line (lower line) and the control line (upper line).

The cross-recognition of both analytes by both aptamers predicted *in silico* was verified in two steps. In the first step, the binding capability of the ampicillin aptamer towards biotinylated CRP was verified, using a lateral flow strip with streptavidin as test line (scheme provided in Electronic Supplementary Material Fig. S2, upper part). Aptamer conjugated gold nanoparticles (AuNPs), generated with the salt aging method were used, as well as FAM-labeled aptamers or scrambled sequences (Electronic Supplementary Material Fig. S3). Aptamers used include the CRP aptamer from Huang *et al*. (α-CRP), the ampicillin aptamer from Song *et al*. (α-Amp-short) and the ampicillin aptamer with an additional 15 nucleotide long thymine spacer at the 5′ end (α-Amp-poly(T)). The additional poly(T) spacer on the ampicillin aptamer was expected to facilitate target access for binding. In Fig. 2, it is shown that all three conjugates bind CRP (CRP, 40.5 µg per strip), but not to BSA (BSA, 40.5 µg per strip). Furthermore, no test line is generated in case of running buffer addition (N). Actually, these results confirm the cross-recognition of CRP by the “ampicillin selective” aptamer. Our data further indicate that the conjugates are not binding to the streptavidin itself by using the no target control (N). Possible electrostatic adsorption of proteins onto the conjugates was excluded by using biotinylated BSA (BSA). We found the test line intensity of α-Amp-poly(T) and α-Amp-short being stronger when compared to α-CRP conjugates, an observation which can be explained by different affinities of the aptamers towards CRP, leading to the conclusion that the ampicillin aptamer owns a higher affinity towards CRP than the CRP aptamer itself.

**Figure 3:**
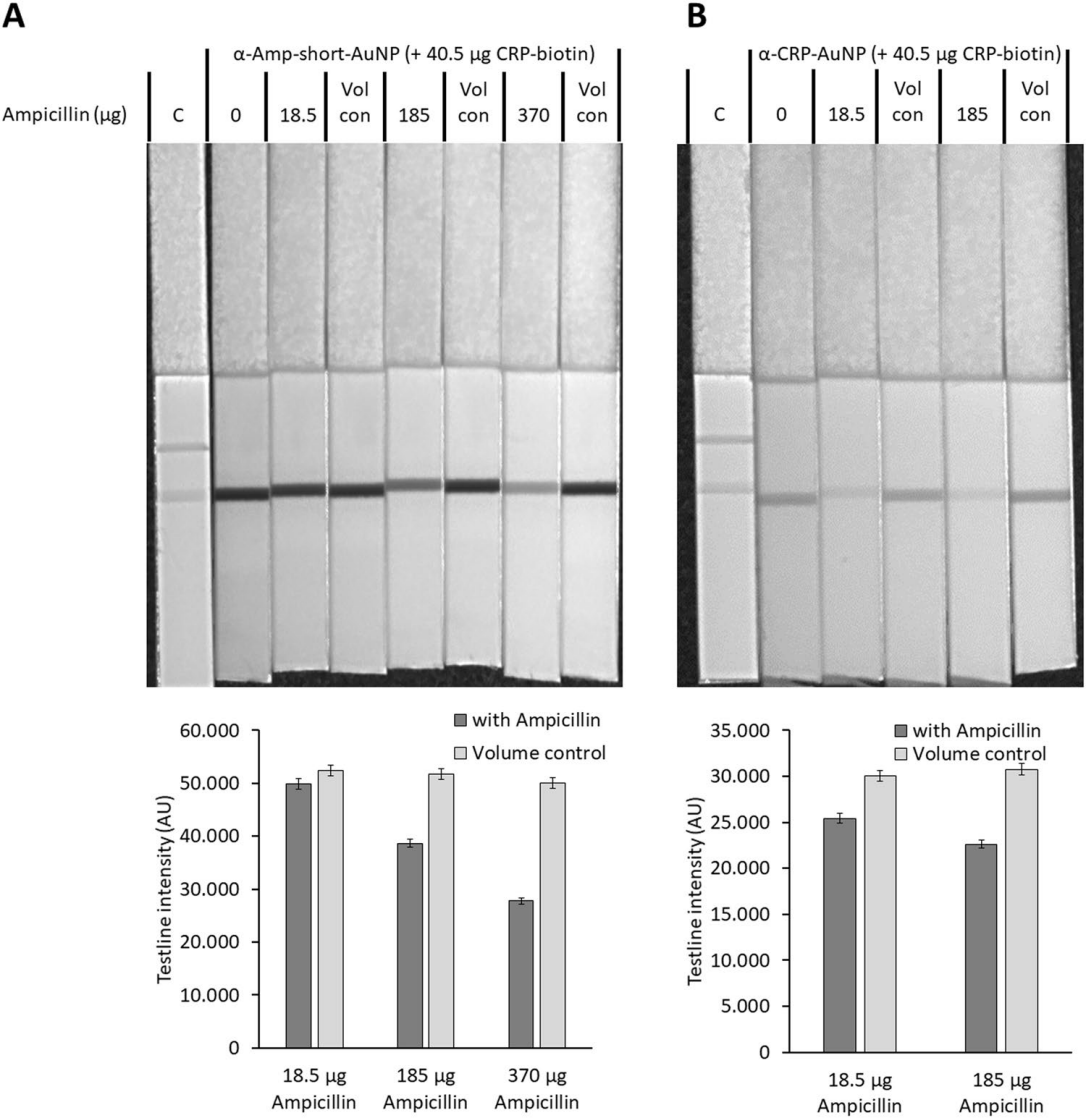
Confirmation of competition between ampicillin and biotinylated CRP using the ampicillin aptamer (**A**) and the CRP aptamer (**B**). A corresponding graph of the test line intensities is shown below each picture. A constant amount of CRP-biotin (40.5 µg) was added to each test stripe with raising amounts of ampicillin (18.5, 185 and 370 µg) and the corresponding amount of binding buffer (Vol con) to verify volume depended effects. Intensity measurement of the test line was done using ImageJ.

In a second step, the binding capability of both aptamers towards ampicillin, as well as the competition of both molecules on the aptamers were investigated (scheme provided in Electronic Supplementary Material Fig. S2, lower part). To this end, a constant amount of biotinylated CRP (40.5 µg per strip) as well as increasing amount of 18.5 mg/mL ampicillin (18.5, 185 and 370 µg per strip) was added to the test stripes as shown in Fig. 3. The same volume of running buffer without ampicillin was added to the test strips, to investigate possible volume related effects. The data show, that in the case of the ampicillin aptamer (α-amp-short-AuNP, Fig. 3A), as well as in the case of the CRP aptamer (α-CRP-aptamer, Fig. 3B) an ampicillin concentration related decrease of the test line intensity is detectable, an effect not related to any volume changes. We thus could demonstrate that both aptamers bind towards CRP as well as ampicillin. The binding of both molecules to the aptamers is apparently competitive; however, as shown in Fig. 3, the amount of biotinylated CRP added, as well as the amount of ampicillin required for a detectable signal decrease at 185 µg ampicillin per test stripe for the ampicillin aptamer and at 18.5 µg ampicillin for the CRP aptamer is quite high. Nevertheless, these results provided insight into the binding affinities of the aptamers. In the case of the ampicillin aptamer, about 326-fold excess of ampicillin is needed to displace sufficient CRP from the aptamer, for obtaining a strong signal decrease. In the case of the CRP aptamer, a 32.6-fold excess of ampicillin already results in a strong signal decrease. This can be due to several factors: firstly, the affinity of both aptamers towards CRP might differ, resulting in altered displacement efficiency of ampicillin. This conclusion is supported by the results of Fig. 2, showing the stronger signal intensity, therefore, the higher affinity in case of the ampicillin aptamers. The second factor is the affinity of both aptamers to ampicillin. If the affinity for ampicillin increases, the displacement efficiency will also increase and thus a reduced signal strength occurs.

Binding of aptamers towards both targets could also be verified by MicroScale Thermophoresis (MST, see Electronic Supplementary Material Fig. S4), showing K_D_ values of 263 and 600 nM, respectively, for ampicillin aptamer binding to ampicillin while also indicating binding in the other cases. These data support our results obtained with other methods and, compared to the CRP aptamer, indicate higher affinity of the ampicillin aptamer to ampicillin.

### Developing a competitive LFA for ampicillin detection

**Figure 4:**
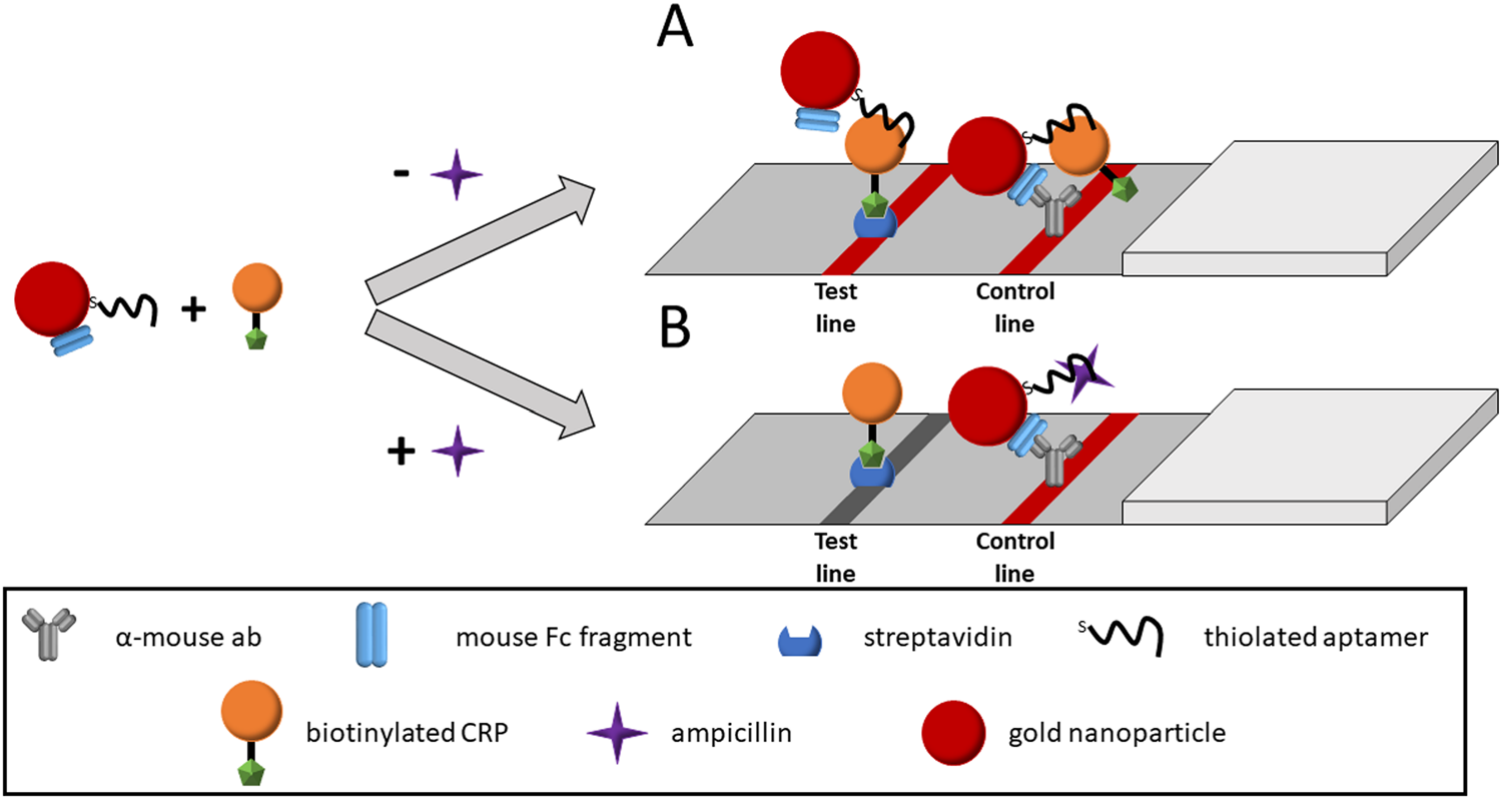
Schematic illustration of the designed competitive LFA for ampicillin detection. Aptamer-mFc-AuNP conjugates are incubated with biotinylated CRP in the presence or absence of ampicillin. If ampicillin is absent, the conjugates will bind towards biotinylated CRP and will be immobilized on the test line with streptavidin (**A**). If ampicillin is present, CRP is displaced from the aptamer, leading to a signal decrease on the test line (**B**). In both cases, the mFc on the conjugates leads to an immobilization on the control line via the α-mouse antibody, generating the control line.

Since the cross-recognition of both molecules by both aptamers could be demonstrated, we further investigated the possibility using this cross-recognition for the development of a LFA for label-free ampicillin detection. Therefore, we designed a test setup as shown in Fig. 4. In this system, AuNPs have been dually functionalized with aptamer and mouse Fc fragment (mFc); biotinylated CRP was added as a competitive agent. If no ampicillin is present, aptamers on the AuNPs bind to the CRP, resulting in a strong test line signal. Unbound probes flow further through the strip and are immobilized on the control line due binding of the mFc to the α-mouse antibodies, resulting in a reliable control line. If ampicillin is present in the sample, it competes with the CRP at the aptamer, resulting in a decreased signal on the test line.

**Figure 5:**
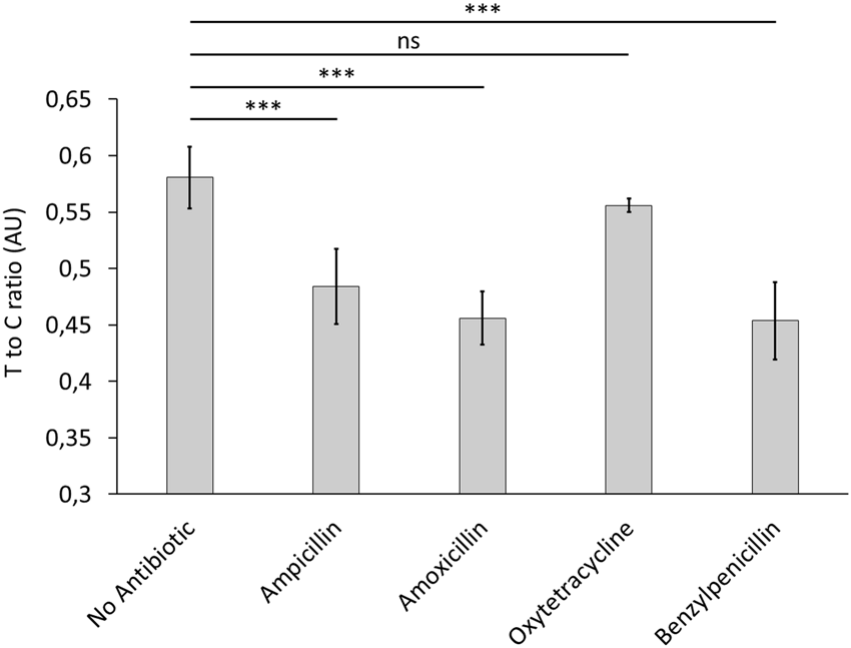
Competitive LFA to verify the selectivity among other antibiotics. 21.17 nmole of each antibiotic was added to each test stripe. The test line to control line intensity (Intensity tl/cl) is given, black bars represent the standard deviation. Each test was performed in triplicates, significance was assessed using Student’s t-test (*P < 0.05; **P < 0.01; ***P < 0.001; ns, not significant).

Nevertheless, as the mouse Fc fragment may have an influence on selectivity and sensitivity of the designed assay, we investigated the effect of amoxicillin, oxytetracycline and benzylpenicillin. Since Song *et al*. reported a high selectivity of one of the ampicillin aptamers towards ampicillin, we expected little to no cross-reaction; however, as shown in Fig. 5, the addition of amoxicillin and benzylpenicillin lead to a significant signal reduction on the test line (ampicillin P value = 0.0013; amoxicillin P value = 0.0002; benzylpenicillin P value = 0.0001). The difference between ampicillin, amoxicillin and benzylpenicillin, however, was not significant. Addition of oxytetracycline does not result in a significant decrease of the test to control ratio (P value = 0.287).

Despite the low specificity towards structural closely related beta lactam antibiotics, we further optimized the assay design for ampicillin detection. As shown in Fig. 3, the amount of ampicillin needed was high in the beginning, preventing use of the test under realistic conditions. As competition of CRP and ampicillin is mainly driven by the ratio of both molecules, we assumed that by lowering the amount of biotinylated CRP used, the amount of ampicillin required for a detectable signal decrease may be reduced. Furthermore, since the number of aptamers bound to biotinylated CRP played a major role during the competition, the overall amount of used aptamer-mFc-AuNP conjugates was diminished.

**Figure 6:**
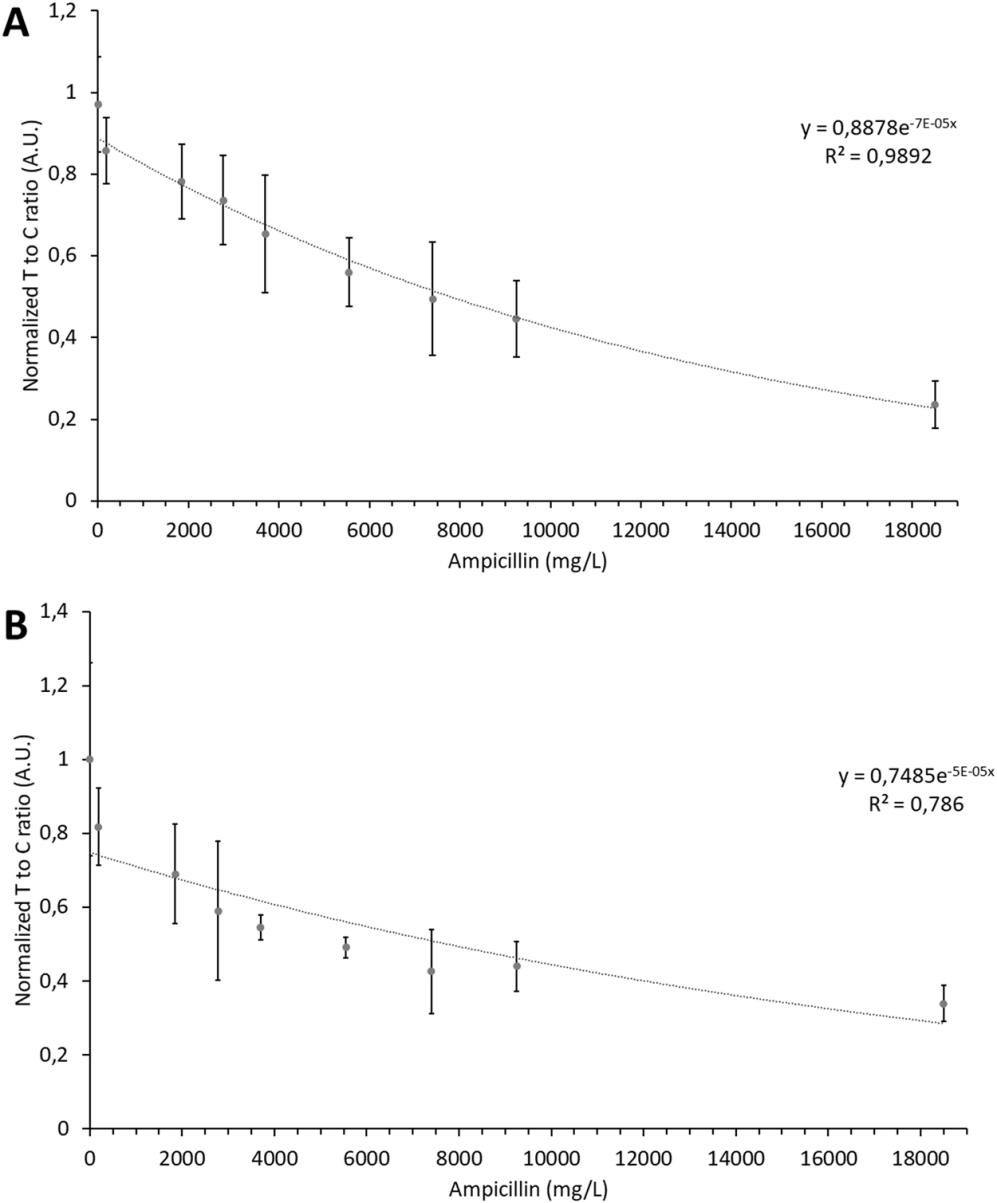
Test line to control line intensity (Intensity tl/cl), normalized to the blank value (no ampicillin addition), in presence of raising ampicillin concentrations in running buffer (**A**) and in milk extract (**B**). Error bars represent the SD of normalized data from four technical replicates, each performed in triplicates (n = 12).

It must be considered, however, that CRP and AuNP conjugate concentrations are both essential for the generation of a strong and reliable test line. Based on our results, the lowest reliable detectable amount of biotinylated CRP was 0.1 µg (4 pmole) and the lowest applicable amount of aptamer-mFc-AuNP conjugates was 0.1 pmole (results not shown). Assuming that a 32.6-fold excess of ampicillin would lead to a significant decrease in signal strength, the theoretical detection limit for ampicillin should be approximately 130 pmole (45.4 ng per strip). However, as shown in Fig. 6A, it was not possible to reduce the detection limit of ampicillin below 10.6 nmole (3.7 µg per strip, P value = 0.01 compared to blank). This is equivalent to an ampicillin concentration of 185 mg/L, if a sample volume of 20 µL is applied. Despite the high detection limit, the test to control line ratio follows a clear trend with increasing ampicillin amounts, as verified by four technical replicates each performed in triplicates.

### Ampicillin detection in real milk sample

We chose milk as analyte matrix to show that the assay is applicable for real sample analysis, even though the detection limit of the designed assay is not yet optimized. Since milk contains biotin which might interfere with biotinylated CRP, we were not able to detect CRP-biotin in non-treated milk (Electronic Supplementary Material Fig. S5, strip 3). Furthermore, we investigated acetic acid treatment of milk according to^27^; even though CRP-biotin could be detected on the test line (Electronic Supplementary Material Fig. S5, strip 2), no signal decrease by ampicillin addition could be observed (results not shown). Thus, we applied a simple extraction of ampicillin from the milk: milk was preincubated with streptavidin to bind free biotin and then filtered through a centrifugal concentrator with 5.000 MWCO. Using this approach, the layer of fat as well as excess of proteins and biotin was removed and CRP-biotin as well as ampicillin could be detected (Electronic Supplementary Material Fig. S4, strip 1).

As shown in Fig. 6B the test line to control line ratio corresponds to raising ampicillin concentrations in the milk. The standard deviation is, compared to the measurement in running buffer, somewhat higher. This effect is likely due to the sample matrix. Since the milk used contained around 1.2 g/L Ca^2+^ and aptamers conformations and thus binding properties heavily depend on the concentration of positive ions, it was assumed that this was one responsible factor. The amount of streptavidin added might have been insufficient to remove all biotin from the sample, leading to a competition of free biotin and biotinylated CRP on the test line. In fact, a lower intensity of the test line was generally observed in case of milk as matrix, compared to experiments using buffer. Due to the high standard deviation, the detection limit lowers. First significant differences were detected at 1850 mg/L ampicillin (P value = 0.0096), also due to the high standard deviation in this case. An evaluation of the extraction efficiency of ampicillin by this method revealed a constant loss of around 260 mg/l ampicillin (likely due to absorbed material), indicating that the weaker signal observed, as well as the signal at 185 mg/l ampicillin (lacking statistical significance), is related to the loss of analyte (see Electronic Supplementary Material Fig. S6). A concentration-dependent effect is, however, still evident, enabling the quantification of ampicillin in milk.

## Discussion

As described, detection of small molecules using LFAs remains a challenge. Since all typical formats of LFA have several core issues for a successful adaption to low molecular weight compounds, we searched for an alternative way of implementing small molecule quantification into LF setups. The typical setup for detection of molecules, which do not exhibit two binding sites, is the competitive LF format. Accordingly, we used this format as starting point and ampicillin as an example. The direct immobilization of ampicillin on a nitrocellulose membrane is quite complex and unfavorable binding, leading to blocked recognition by the aptamer has to be expected; therefore, the biotin streptavidin system was chosen for target immobilization. We, however, failed on detecting biotinylated ampicillin, likely due to the modified structure of ampicillin after biotinylation (results not shown). Aptamers never exhibit absolute target selectivity. As immobilization of the target itself failed, the immobilization of a cross-recognized molecule was therefore considered. Since a high selectivity of the aptamer with regard to other beta-lactams was reported by Song *et al*.^23^, we considered the use of proteins for cross-recognition instead of other beta lactam antibiotics. The usage of proteins as counter-selective agents during the SELEX process of small molecule aptamer, however, is to our knowledge so far not described.

We therefore, searched for a possible selective cross-recognition by comparing several DNA aptamer sequences. As demonstrated, this approach might be most suitable for an *in-silico* identification of cross recognitions, assuming that, if different aptamers share partial sequences, they might also bind to identical targets.

In fact, we could experimentally confirm the predicted cross-recognition of the ampicillin aptamer with CRP and *vice versa* for the CRP aptamer and were able to use this cross-reaction for the development of a LFA for ampicillin detection. The binding of both aptamers to both targets was also verified via MST measurements. This, in fact, denotes a major progress given the failure of previous approaches in our laboratory for developing a LFA for biotinylated ampicillin and lack of success may most likely be attributed to the conformational changes of the ampicillin after biotinylation with a NHS-biotin reagent.

At current, no statement on the affinity of both aptamers towards ampicillin can be made; however, since the ratio of the affinity towards ampicillin and the affinity towards CRP is the pivotal factor for this competition, it can be concluded that the affinity ratio of the CRP aptamer (compared to the ampicillin aptamer) is shifted towards ampicillin. It should be mentioned, that the target molecular weight is proportional to the resulting aptamers affinity and therefore, it is not surprising that an excess of ampicillin for CRP displacement from the aptamer is needed in all cases^19^.

As a competitive displacement is possible, our results indicate that both aptamers bind using the same nucleotide sequences. This also suggests that both molecules share similar binding sites for the aptamers. Since it has been reported that the ampicillin aptamer binds towards the side chain of ampicillin, it has been assumed that the site being bound consists of a benzene ring with an nearby amine group^23^. On the polypeptide level, this structure might be imitated by steric proximity of the side chain of a phenylalanine (bearing a phenyl substituent) with an arginine or lysine (bearing amine groups in the side chain). Further experiments, however, should be performed to give insights into the binding mechanism of the aptamers towards CRP.

As the mouse Fc fragment may have an influence on selectivity and sensitivity of the assay, we examined the effect of amoxicillin, oxytetracycline and benzylpenicillin. The difference between ampicillin, amoxicillin and benzylpenicillin, however, was not significant and addition of oxytetracycline did not result in a significant increase of signal strength. These results indicate a low specificity towards ampicillin, likely attributable to the low specificity of the aptamer itself. Buffer conditions can play a role for the specificity^28^. Molecular docking, however, has shown that lidocaine binds towards CRP using three different binding sites^29^. One binding site is a π-π interaction between the aromatic moiety of lidocaine with Phe66 with the lidocaine-CRP interaction leading to a lowered binding probability of an aptamer^29^. This interaction may hold true for beta lactam antibiotics as well, since they have an aromatic moiety in the side chain. Li *et al*., however, used another aptamer and interaction was only observed after an incubation time exceeding one hour, making an effect from the interaction between the beta lactams and CRP quite unlikely. No information was, however, found, regarding the interaction of beta lactam antibiotics and CRP.

The high detection limit for ampicillin might be mediated by several factors: the affinity of CRP towards the aptamer seems to be higher than the affinity of ampicillin, as previously mentioned. Furthermore, since CRP is larger than ampicillin, CRP might lower the binding probability of ampicillin-aptamer due to steric hindrance after binding. The assay format may also have an influence: since CRP is immobilized on the test line with biotin, the local concentration of CRP rises, whereas, the local concentration of ampicillin remains constant. We assume that competition of both molecules takes place during migration of the strip and thus, is strongly shifted towards CRP at the test line. This problem might be addressed by immobilizing the CRP onto the AuNPs and the aptamer onto the test line. However, due to afore mentioned factors a lower detection limit of ampicillin might be unachievable with the given assay format; other assay formats, like ELISA, may, however, well achieve lower detection limits.

We here report the cross-recognition of CRP and ampicillin by CRP and ampicillin aptamers, identified by *in silico* analysis of sequence homologies of both aptamers. We could also show that this cross-recognition is following a competitive binding scheme we translated to a LFA. The assay design is based on a strip with streptavidin on the test line and α-mouse antibodies on the control line. AuNPs were dually-functionalized with mouse Fc fragment and the aptamer, then incubated with biotinylated CRP and ampicillin. A concentration dependent detection of ampicillin could be achieved, specificity towards beta lactams, as well as sensitivity, however, remained low requiring further optimization; however, we could demonstrate that the test system can be applied for real sample matrix analysis, showing general feasibility of such formats.

Cross-recognition has been described here and applied for small molecule analysis in a LF-format for the first time. Despite applicability of the identified cross-recognition for real sample analysis, it should be noted that such cross-recognitions involving other, unconsidered targets might also interfere with desired applications of such aptamer-based assays. The main point, however, lies in the potential to transfer this setup to almost any small molecule target rendering LFA feasible for such target molecules, *e*.*g*. for small endogenous metabolites gaining increasing relevance as diagnostic biomarker. Using known aptamers or novel sequences identified by *in-vitro* selection in combination with subsequently selected competing immunogenic peptides, e.g. selected by powerful peptide library screening (which has been shown to be successfully applied for tumor-targeting^30^ or antimicrobial peptides^31^), would not only identify more such cross-recognitions but also result in a high affinity of the aptamer towards both targets, fostering practical applications. Almost any small molecule target may thus be addressed by an LF-assay applying the presented approach (without optimizing conditions in the very case presented) offering, however, a transferable concept for LAF assays for small molecules.

## Methods

### Reagents and materials

Gold nanoparticles (AuNPs), 9.46 nM 10 nm diameter, were purchased from BBI Solutions (Cardiff, UK). NHS-PEG4-Biotin was purchased from Thermo Fisher Scientific, Inc. (Waltham, MA). C-reactive protein (CRP) was purchased from Innovative Research (Novi, MI). Ampicillin was purchased from Carl Roth GmbH (Karlsruhe, Germany). Mouse Fc fragment (mFc) was purchased from Jackson ImmunoResearch Europe Ltd. (Newmarket, UK). Tris-acetate-EDTA buffer (40 mM Tris, 20 mM acetic acid, 1 mM EDTA), sodium chloride, magnesium chloride, calcium chloride, tris(2-carboxyethyl)phosphine (TCEP), streptavidin and all other reagents where purchased from Sigma-Aldrich (Munich, Germany) and were at least of analytical grade. Lateral Flow Strips were prefabricated by R-Biopharm AG (Darmstadt, Germany), with Streptavidin on the test line and anti-mouse antibody from goat on the control line. Pur-A-Lyzer Dialysis Kits, 10 kDa MWCO, were from Sigma-Aldrich (Munich, Germany). 5,000 MWCO centrifugal concentrators were from Sartorius (Goettingen, Germany). Oligonucleotides were from Integrated DNA Technology (Coraville, IA), sequences are as follows:

α-Amp-short: 5′-SH-(C6H12)-CAC GGC ATG GTG GGC GTC GTG-3′

α-Amp-pol(T): 5′-SH-(C6H12)-TTT TTT TTT TTT TTT CAC GGC ATG GTG GGC GTC GTG-3′

α-CRP: 5′-SH-(C6H12)-ACA CGA TGG GGG GGT ATG ATT TGA TGT GGT TGT TGC ATG ATC GTG G-3′

### Instrumentation and software

All solutions were prepared in high-purity water obtained from a Barnstead MicroPure ST System from Thermo Fisher Scientific, Inc. (Waltham, MA). Digital pictures were obtained using ChemStudio Plus from Analytik Jena (Jena, Germany). The Mfold server located http://mfold.bioinfo.rpi.edu/ was used to model the folding form of the oligonucleotides in the presence of 55 mM Na^+^ and 7 mM Mg^2+^ at 20 °C. For the intensity measurement of the lines, the imaging software ImageJ, available at the National Institutes of Health (NIH) website, was used.

### Aptamer functionalized gold nanoparticles

Salt aged aptamer-AuNP conjugates were prepared according to a reported method^2^. Thiolated oligonucleotides were activated by using a 2-fold excess of TCEP in 50 mM citrate buffer (pH 7) for an incubation of 1 h at RT. Briefly, 100 µL of 25 µM activated aptamer solution was mixed with 500 µL of 9.46 nM AuNP solution and incubated for 16 h under dark conditions under tilt rotation at RT. Afterwards, 5.44 µL of 500 mM TAE (pH 8.2) and 60 µL of 1 M NaCl was slowly added (6 µL each 20 min), followed by 24 h incubation under dark conditions under tilt rotation at RT. After incubation, the conjugates were centrifuged at 14,100 rcf for 45 min, the supernatant was removed and the pellet resuspended in 500 µL ddH_2_O, following another centrifugation at 14,100 rcf for 90 min and finally resuspended in 47.1 µL aptamer binding buffer (20 mM Tris, 50 mM NaCl, 5 mM KCl, 5 mM MgCl_2_, 2 mM CaCl_2_, pH 8), resulting in an approximately 100 nM aptamer-AuNP solution (respective to the AuNP concentration). Surface functionalization was confirmed via UV-Vis measurements (Electronic Supplementary Material Fig. S1) prior to use.

### Aptamer mouse Fc fragment functionalized gold nanoparticles

Salt aged aptamer-mFc-AuNP conjugates were prepared according to the modified method from^2^. Thiolated oligonucleotides were activated by using a 2-fold excess of TCEP in 50 mM citrate buffer (pH 7) for an incubation of 1 h at RT under tilt rotation. Activated aptamer solution then was mixed with mouse Fc fragment, yielding a final aptamer concentration of 10 µM and 0.5 µM mFc in 100 µL. The mixture was then added to 500 µL of 9.46 nM AuNP solution following an incubation for 16 h in dark conditions under tilt rotation at RT. Conjugation then proceeded as described above, resulting in an approximately 100 nM aptamer-mFc-AuNP solution in aptamer binding buffer. Surface functionalization was confirmed via UV-Vis measurements (Electronic Supplementary Material Fig. S1) prior to use.

### Biotinylation of CRP

Biotinylation of CRP was achieved by using NHS-PEG4-Biotin. 990 µL of 2 mg/mL CRP was mixed with 10 µL of 33.97 mM NHS-PEG4-Biotin and incubated for 5 h at RT on a horizontal shaker (1000 rmp). Afterwards, the mixture was dialyzed, using Pur-A-Lyzer Dialysis Kits (10 kDa MWCO), against 100 mM Tris, 200 mM NaCl, 2 mM CaCl_2_, pH 7.5, diluted 10-fold in binding buffer and stored at 4 °C until further use.

### Lateral Flow Assay

A volume of 40 µL running buffer (20 mM Tris, 50 mM NaCl, 5 mM KCl, 5 mM MgCl_2_, 2 mM CaCl_2_, 0.1 mM BSA, 1.7% Triton-X-100, pH 8) was mixed with 1 µL of 0.2 mg/mL biotinylated CRP and 20 µL of analyte solution. Afterwards, 1 µL of the aptamer-AuNP or aptamer-mFc-AuNP conjugates were added to the mixture and incubated for 20 min. The mixture then was allowed to flow through the lateral flow strip for 10 minutes and pictures were taken. Test line to control line intensity ratio (Intensity tl/cl) was assessed by dividing the test line intensity through the control line intensity.

### Preparation of real milk sample

Milk (3.5% fat) was bought from a local supermarket. Briefly, 200 µL of the milk was spiked with different concentration of antibiotics and mixed with 20 µL of 1 mg/mL streptavidin. After incubation for 20 minutes on a horizontal shaker (600 rpm) at RT, the mixture was filtered through a centrifugal concentrator (5.000 MWCO) at 15,000 rcf for 80 min at 4 °C. The filtered solution was stored at 4 °C until further use.

### Statistics and bioinformatics

Statistical analysis, as well as sequence comparison was performed by using the statistical software R^32^. Technical replicates were normalized by dividing the intensity ratio tl/cl by the mean of the blank. If not stated otherwise, mean and SD are reported. Significance was assessed by a one-way ANOVA with dummy coding, where a p value smaller than 0.05 for the dummy variables was considered as significant. In addition, a simple exponential model was used for fitting trend lines.

## Electronic supplementary material

Supplemental material

## References

[1] Posthuma-Trumpie GA, Korf J, Van Amerongen A (2009). Lateral flow (immuno)assay: Its strengths, weaknesses, opportunities and threats. A literature survey. Anal. Bioanal. Chem..

[2] Jauset-Rubio M (2016). Aptamer Lateral Flow Assays for Ultrasensitive Detection of β-Conglutin Combining Recombinase Polymerase Amplification and Tailed Primers. Anal. Chem..

[3] Chen A, Yang S (2015). Replacing antibodies with aptamers in lateral flow immunoassay. Biosens. Bioelectron..

[4] Gonzalez JM, Foley MW, Bieber NM, Bourdelle PA, Niedbala RS (2011). Development of an ultrasensitive immunochromatography test to detect nicotine metabolites in oral fluids. Anal. Bioanal. Chem..

[5] Liu Y (2015). Detection of 3-phenoxybenzoic acid in river water with a colloidal gold-based lateral flow immunoassay. Anal. Biochem..

[6] Chen Y (2016). Near-infrared fluorescence-based multiplex lateral flow immunoassay for the simultaneous detection of four antibiotic residue families in milk. Biosens. Bioelectron..

[7] Xu H (2009). Aptamer-functionalized gold nanoparticles as probes in a dry-reagent strip biosensor for protein analysis. Anal. Chem..

[8] Bruno J (2014). Application of DNA Aptamers and Quantum Dots to Lateral Flow Test Strips for Detection of Foodborne Pathogens with Improved Sensitivity versus Colloidal Gold. Pathogens.

[9] Liu G (2009). Aptamer−Nanoparticle Strip Biosensor for Sensitive Detection of Cancer Cells. Anal. Chem..

[10] Zhang Q (2016). Aptamer-based Dry-reagent Strip Biosensor for Detection of Small Molecule ATP. Chem. Lett..

[11] Özalp VC (2016). Small molecule detection by lateral flow strips via aptamer-gated silica nanoprobes. Analyst.

[12] Fischer C, Wessels H, Paschke-Kratzin A, Fischer M (2017). Aptamers: Universal capture units for lateral flow applications. Anal. Biochem..

[13] Liu J, Mazumdar D, Lu Y (2006). A simple and sensitive ‘dipstick’ test in serum based on lateral flow separation of aptamer-linked nanostructures. Angew. Chemie - Int. Ed..

[14] Mazumdar D, Liu J, Lu G, Zhou J, Lu Y (2010). Easy-to-use dipstick tests for detection of lead in paints using non-cross-linked gold nanoparticle–DNAzyme conjugates. Chem. Commun..

[15] Wang L (2011). Fluorescent strip sensor for rapid determination of toxins. Chem. Commun..

[16] Shim WB, Kim MJ, Mun H, Kim MG (2014). An aptamer-based dipstick assay for the rapid and simple detection of aflatoxin B1. Biosens. Bioelectron..

[17] McKeague M (2015). Analysis of *In Vitro* Aptamer Selection Parameters. J. Mol. Evol..

[18] Chen J, Fang Z, Lie P, Zeng L (2012). Computational lateral flow biosensor for proteins and small molecules: A new class of strip logic gates. Anal. Chem..

[19] McKeague, M. & Derosa, M. C. Challenges and opportunities for small molecule aptamer development. *J*. *Nucleic Acids* **2012**, (2012). 10.1155/2012/748913 PMC3488411 23150810

[20] Wei C-W, Cheng J-Y, Huang C-T, Yen M-H, Young T-H (2005). Using a microfluidic device for 1 l DNA microarray hybridization in 500 s. Nucleic Acids Res..

[21] Kaushik D, Mohan M, Borade DM, Swami OC (2014). Ampicillin: Rise fall & resurgence. J. Clin. Diagnostic Res..

[22] Campagna JD, Bond MC, Schabelman E, Hayes BD (2012). The Use of Cephalosporins in Penicillin-allergic Patients: A Literature Review. J. Emerg. Med..

[23] Song KM, Jeong E, Jeon W, Cho M, Ban C (2012). Aptasensor for ampicillin using gold nanoparticle based dual fluorescence-colorimetric methods. Anal. Bioanal. Chem..

[24] Andreou C, Mirsafavi R, Moskovits M, Meinhart CD (2015). Detection of low concentrations of ampicillin in milk. Analyst.

[25] Huang CJ, Lin HI, Shiesh SC, Lee G (2010). Bin. Integrated microfluidic system for rapid screening of CRP aptamers utilizing systematic evolution of ligands by exponential enrichment (SELEX). Biosens. Bioelectron..

[26] Stewart S (2011). Identifying protein variants with cross-reactive aptamer arrays. ChemBioChem.

[27] Lee A-Y (2017). Development of a ssDNA aptamer for detection of residual benzylpenicillin. Anal. Biochem..

[28] Jeong S, Rhee Paeng I (2012). Sensitivity and Selectivity on Aptamer-Based Assay: The Determination of Tetracycline Residue in Bovine Milk. Sci. World J..

[29] Li Z (2017). Evaluating the Effect of Lidocaine on the Interactions of C-reactive Protein with Its Aptamer and Antibody by Dynamic Force Spectroscopy. Anal. Chem..

[30] Liu R, Li X, Xiao W, Lam KS (2017). Tumor-targeting peptides from combinatorial libraries. Adv. Drug Deliv. Rev..

[31] Ashby M, Petkova A, Gani J, Mikut R, Hilpert K (2017). Use of Peptide Libraries for Identification and Optimization of Novel Antimicrobial Peptides. Curr. Top. Med. Chem..

[32] R Core Team. *R: A language and enviroment for statistical computing*. (R Foundation for Statistical Computing, 2017).

