# Supplementary material for: Small molecule detection with aptamer based lateral flow assays: Applying aptamer-C-reactive protein cross-recognition for ampicillin detection
Source: Sci Rep. 2018 Apr 4;8:5628. doi: 10.1038/s41598-018-23963-6 (PMC5884802; doi:10.1038/s41598-018-23963-6)
Supplement: Supplementary file 1 — Supplemental material [file 41598_2018_23963_MOESM1_ESM.docx]

**Scientific Reports**

**Electronic Supplementary Material**

**Small molecule detection with aptamer based lateral flow assays: Applying aptamer-C-reactive protein cross-recognition for ampicillin detection**

Lars Kaiser^1^, Julia Weisser^1^, Matthias Kohl^1^ and Hans-Peter Deigner^1,2*^

^1^ Furtwangen University, Institute of Precision Medicine, Jakob-Kienzle-Straße 17, 78054 Villingen-Schwenningen, Germany

^2^Fraunhofer Institute IZI, Leipzig, EXIM Department, Schillingallee 68, D-18057 Rostock, Germany

*Corresponding author: Phone: +4977203074232, Fax: +4977203074725, e-mail: hans-peter.deigner@hs-furtwangen.de

**Additional methods:**

**Reagents and Materials**

Lateral Flow Strips were prefabricated by R-Biopharm AG (Darmstadt, Germany), with Streptavidin on the test line and anti-mouse antibody from goat on the control line. Oligonucleotides were from Integrated DNA Technology (Coraville, IA), sequences are as follows:

CRP-FAM: 5´-FAM ACA CGA TGGG GGG GTA TGA TTT GAT GTG GTT GTT GCA TGA TCG TGG-3´

Amp(short)-FAM: 5´-FAM CAC GGC ATG GTG GGC GTC GTG-3´

CRP w/o TGG-FAM: 5´-FAM ACA CGA ACC GGG CCA ATG ATT TGA TGA CCT TGT TGC ATG ATC GTA G-3´

Amp(short) w/o TGG-FAM: 5´-FAM CAC GGC ATA GAC CGC GTC GTG-3´

CRP scrambled-FAM: 5´-FAM CTT ACT AAT TAA AAC TCA TAA ATA CTC TAA TTA CCC GCC TAA TCA A-3´

Amp(short) scrambled-FAM: 5´-FAM TCT AAT ATA ATC TAT ACT CCT-3´

**Characterization of Aptamer-AuNP, Aptamer-mFc-AuNP and mFc-AuNP conjugates**

Surface functionalization of AuNPs was verified via UV-Vis measurements. Briefly, prepared conjugates (~100 nM) were diluted 1:10 in ddH_2_O resulting in 100 µl in total and absorbance between 450-570 nm was measured using a TECAN infinite 200Pro plate reader from Tecan Group Ltd. (Männedorf, Switzerland). Absorbance peaks were normalized to the peak maximum by dividing the absorbance values by the maximum absorbance value.

**Lateral Flow Assay using Aptamer-AuNP conjugates**

A volume of 40 µL running buffer (20 mM Tris, 50 mM NaCl, 5 mM KCl, 5 mM MgCl_2_, 2 mM CaCl_2_, 0.1 mM BSA, 1.7 % Triton-X-100, pH 8) was mixed with 40.5 µg biotinylated Protein or equivalent volume of running buffer. 20 µl of ampicillin in running buffer or just running buffer was added in indicated cases. Afterwards, 1 µL of the aptamer-AuNP conjugates were added to the mixture and incubated for 20 min. The mixture then was allowed to flow through the lateral flow strip and pictures were taken. Test line to control line intensity ratio (Intensity tl/cl) was assessed by dividing the test line intensity through the control line intensity.

**Lateral Flow Assay using FAM-labeled oligonucleotides**

A volume of 40 µL running buffer (20 mM Tris, 50 mM NaCl, 5 mM KCl, 5 mM MgCl_2_, 2 mM CaCl_2_, 0.1 mM BSA, 1.7 % Triton-X-100, pH 8) was mixed with 2 µL of 2 mg/mL biotinylated CRP. Afterwards, 5 µL of 100 µM FAM-labeled aptamers were added to the mixture and incubated for 10 min. The mixture then was allowed to flow through the lateral flow strip and pictures were taken.

**MicroScale Thermophoresis (MST) measurements**

MST measurements were performed by 2bind GmbH Molecular interaction services (Regensburg, Germany), using FAM-labeled oligonucleotides, ampicillin and CRP-biotin. FAM-labeled oligonucleotides were held constant at 5 nM with varying concentrations of analytes, ranging from 3.05 nM – 100 µM for ampicillin and 610 pM – 20 µM for CRP-biotin. The used assay buffer was 20 mM Tris-HCL, 50 mM NaCl, 5 mM KCl, 5 mM MgCl_2_, 2 mM CaCl_2_, 0.1% Tween-20, pH 8.0 for the analyte ampicillin, in case of CRP-biotin as analyte the buffer consisted of 32.8 mM Tris-HCl, 70.5 mM NaCl, 2.5 mM KCl, 2.5 mM MgCl_2_, 1.4 mM CaCl_2_, 0.1% Tween-20. Measurement was performed using a Monolith NT.115 Pico from NanoTemper Technologies GmbH (Munich, Germany).

**Evaluation of different milk pre-treatments for real sample analysis**

Milk (3.5% fat) was bought from a local supermarket and different pre-treatments were applied. Milk was either treated with 5% acetic acid, centrifuged for 15 min at 5.000 rcf at RT and filtered through a 0.22 µm filter according to Lee et al ^1^, or treated with streptavidin and filtered as described in the manuscript. Also, non-treated milk was used. 20 µl of the milk were added to 40 µl of running buffer and 1 µl 0.1 mg/ml CRP-biotin, as well as 1 µl of the α-Amp-short-mFc-AuNP conjugates were added and the mixture was allowed to flow through the lateral flow strip for 10 minutes.

**Ampicillin concentration determination in milk via ELISA**

Ampicillin concentration in milk prior and after the applied extraction method was determined by an independent method using the *RIDASCREEN® Penicillin* from R-Bipharm AG (Darmstadt, Germany) according to the manufacturer’s protocol. Milk was spiked with different concentrations of ampicillin, half of the milk was stored on ice until the analysis and the other half was extracted as described in the manuscript. Pure spiked Milk, as well as spiked milk extract were diluted prior analysis to meet quantification limits of the ELISA.

**Figures:**


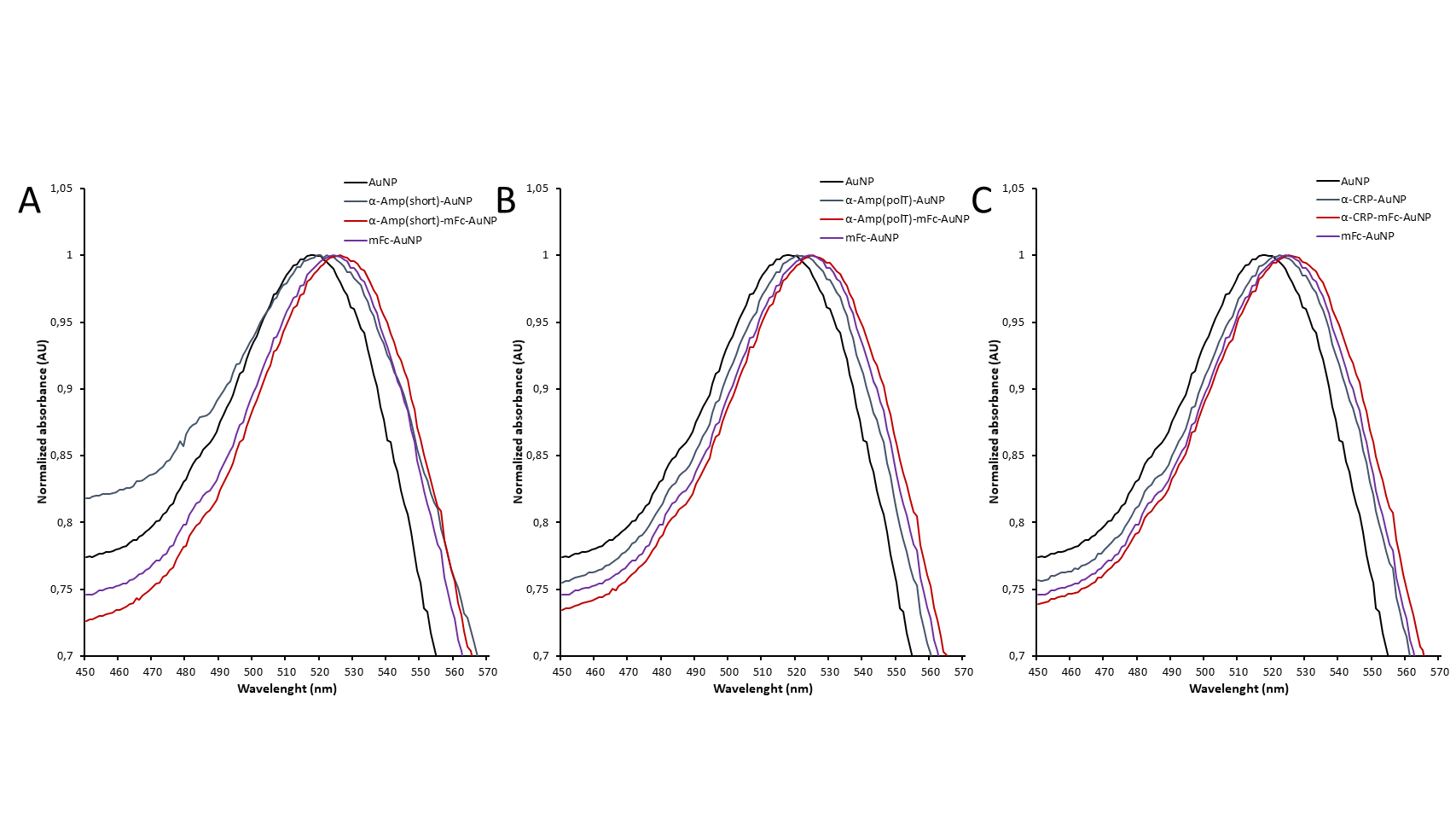


**Fig S1** UV-Vis spectra of citrate-stabilized AuNPs (black), aptamer functionalized AuNPs (grey), aptamer and mFc dual-functionalized AuNPs (red) and mFc functionalized AuNPs (violet). Spectra are shown for the α-Amp(short) aptamer (A), the α-Amp-poly(T) aptamer (B) and the α-CRP aptamer (C). Peak maxima were the following; 517-518 nm for citrate-stabilized AuNPs, 519-522 nm for aptamer functionalized AuNPs, 525-526 nm for aptamer and mFc dual-functionalized AuNPs and 524 nm for mFc functionalized AuNPs.


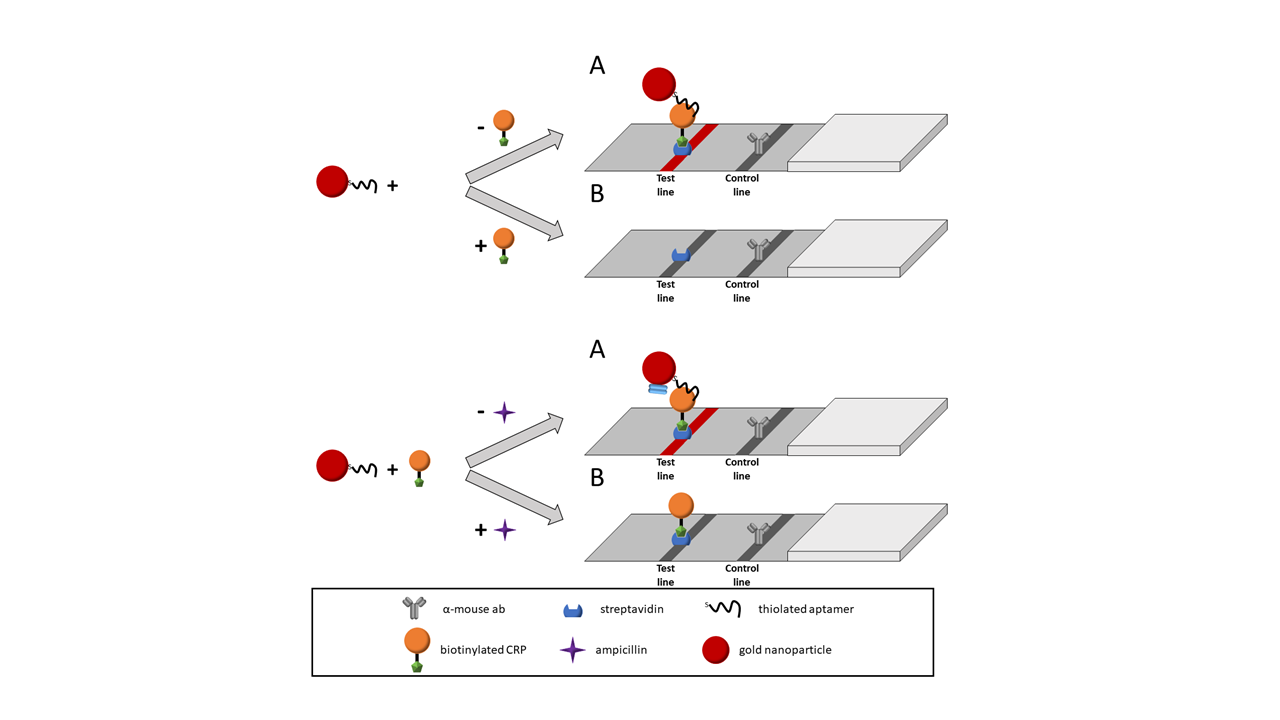


**Figure S2** Schematic display of the Lateral Flow Assay, using aptamer-AuNP conjugates. Conjugate binding towards CRP-biotin was tested as shown in the upper part of the picture, competitive conjugate binding between CRP-biotin and ampicillin was tested as shown in the lower picture.


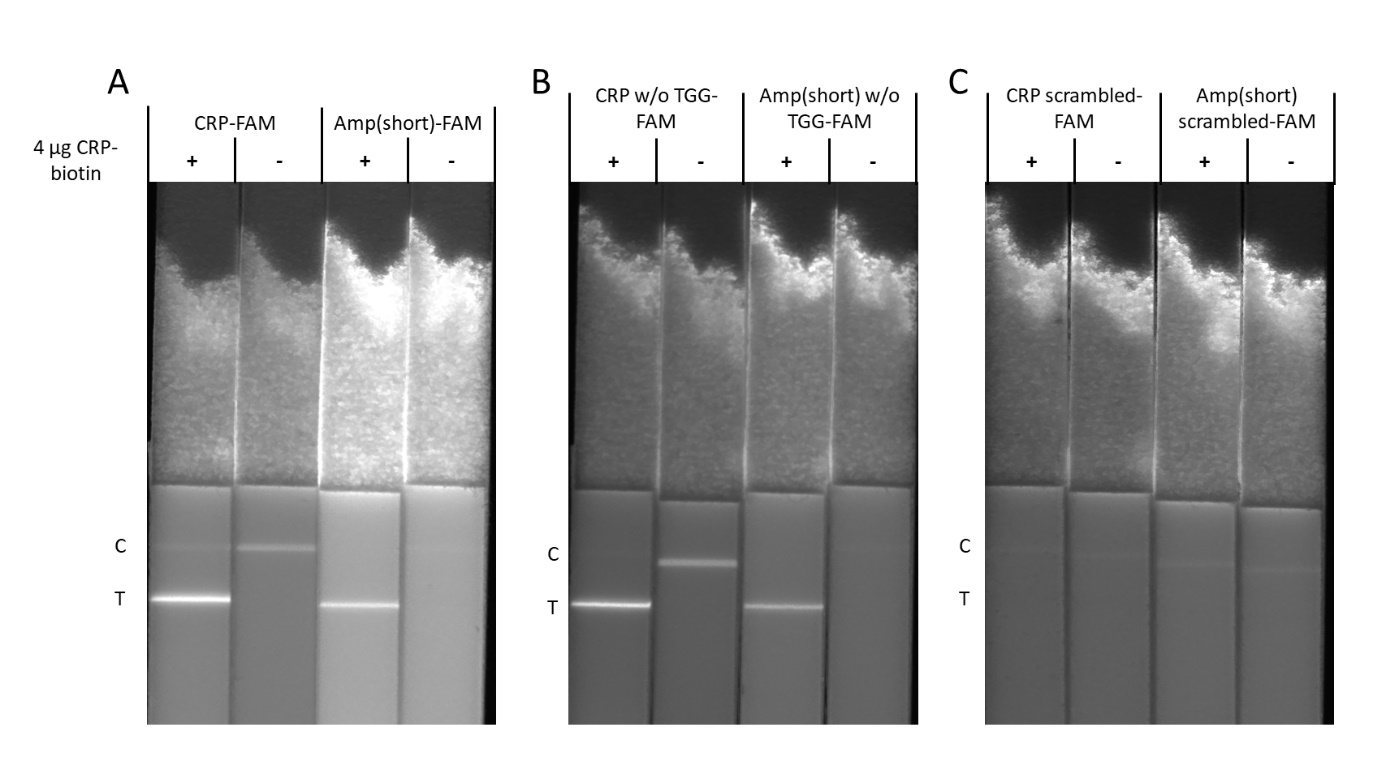


**Figure S3** Lateral Flow Assay, using different FAM-labeled aptamer sequences in presence (+) or absence (-) of 4 µg CRP-biotin. Test strips are shown for the original aptamer sequences (A), mutated sequences lacking the TGG domains (B) and scrambled control sequences (C). T indicates the test line and C the control line.


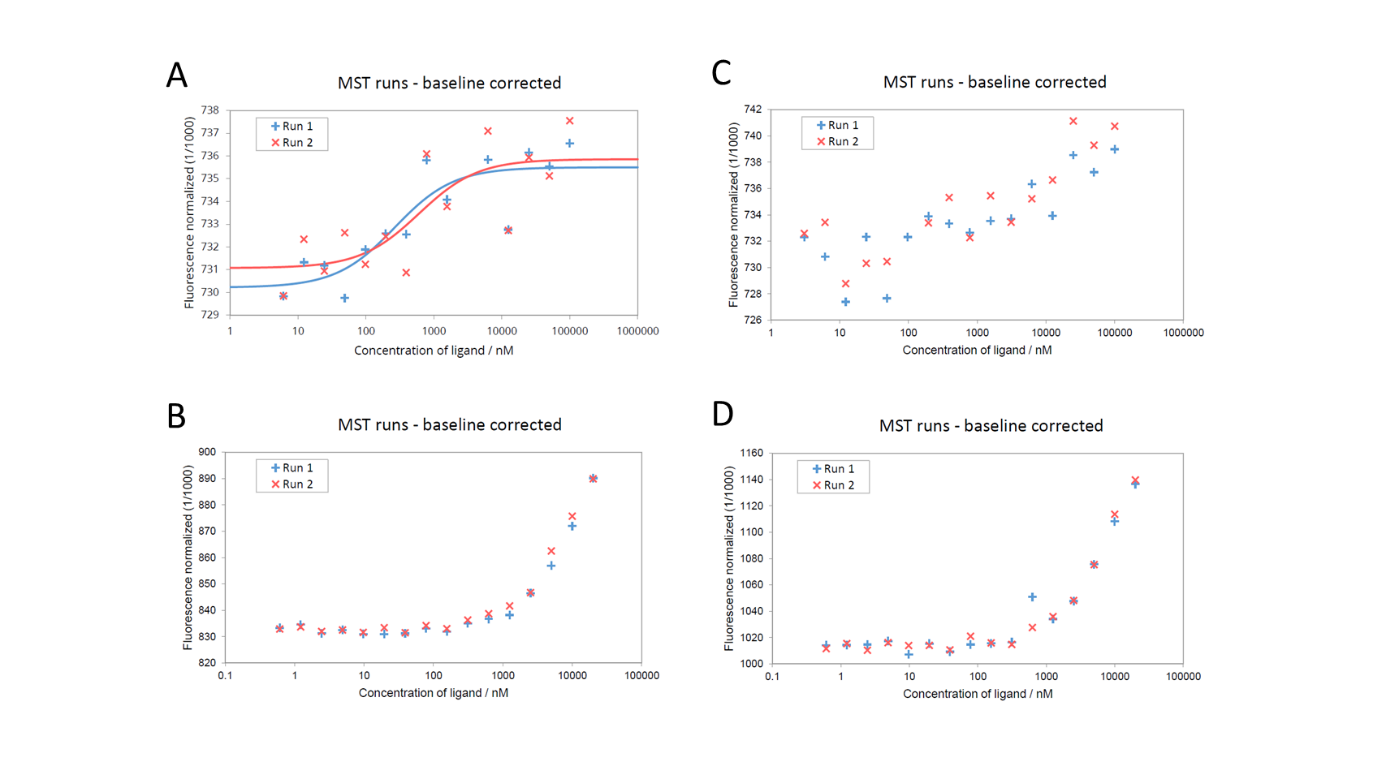


**Figure S4** Fluorescent MicroScale Thermophoresis results of the α-Amp(short) aptamer and the target ampicillin (A), the α-Amp(short) aptamer and the target CRP (B), the α-CRP aptamer and the target ampicillin (C) and the α-CRP aptamer and the target CRP (D). The obtained K_D_ values from (A) are 263 nM and 600 nM respectively, in the other cases binding is indicated but no proper curve fit was possible due to no bound plateau was reached.


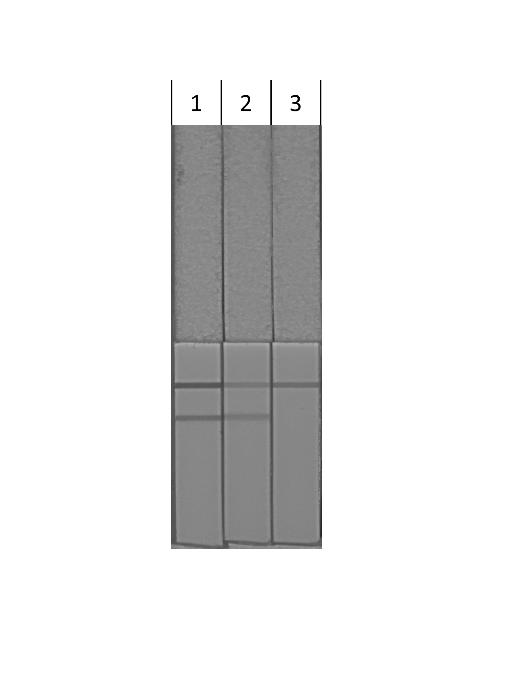


**Figure S5** Different pre-treatment of milk for CRP-biotin detection. A constant amount of CRP-biotin (0.1 µg) was added to each sample and different sample matrices were evaluated; streptavidin treatment and filtration for strip 1, acetic acid treatment for strip 2 and pure milk for strip 3.


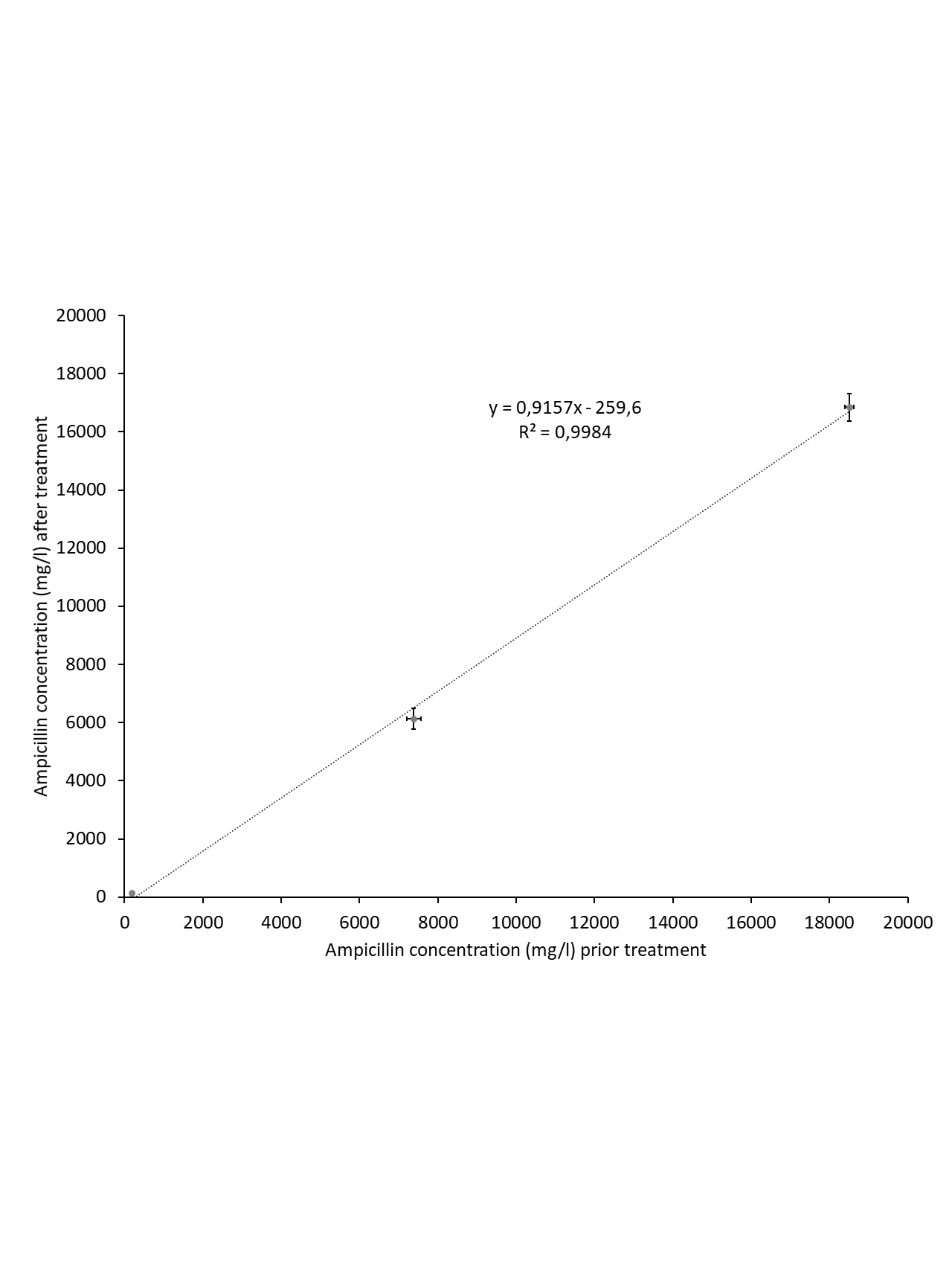
**Figure S6** Ampicillin concentration determination via competitive ELISA in milk prior (x axis) and after (y axis) streptavidin treatment and filtration.

**References:**

1. Lee, A.-Y. *et al.* Development of a ssDNA aptamer for detection of residual benzylpenicillin. *Anal. Biochem.* **531,** 1–7 (2017).
